# Supplementary material for: Quantum spin transistor with a Heisenberg spin chain
Source: Nat Commun. 2016 Oct 10;7:13070. doi: 10.1038/ncomms13070 (PMC5062499; doi:10.1038/ncomms13070)
Supplement: Supplementary Information — Supplementary Notes 1-4 and Supplementary References [file ncomms13070-s1.pdf]

Supplementary Note 1: $N = 3$ spin chain.

Here we find the gate magnetic field h_j required for the realization of the quantum spin transistor in a Heisenberg XXZ spin chain described by the Hamiltonian

$$H = \sum_{j=1}^{N} h_j \hat{\sigma}_z^j - \frac{1}{2} \sum_{j=1}^{N-1} J_j [\hat{\sigma}_x^j \hat{\sigma}_x^{j+1} + \hat{\sigma}_y^j \hat{\sigma}_y^{j+1} + \Delta \hat{\sigma}_z^j \hat{\sigma}_z^{j+1}], \tag{1}$$

with $N = 3$, $h_{1,N} = 0$ and $J_i = J_{N-i}$. We start with the spin chain of one spin-up and two spin-down particles. Our first goal is to transfer the population of the initial state $|\uparrow\downarrow\downarrow\rangle$ to the final state $|\downarrow\downarrow\uparrow\rangle$ via the intermediate state $|\downarrow\uparrow\downarrow\rangle$.

In the basis of $\{|\uparrow\downarrow\downarrow\rangle, |\downarrow\uparrow\downarrow\rangle, |\downarrow\downarrow\uparrow\rangle\}$, the Hamiltonian in Supplementary Equation (1) is

$$H = \begin{pmatrix} -h & -J_1 & 0 \\ -J_1 & h + J_1\Delta & -J_2 \\ 0 & -J_2 & -h \end{pmatrix}, \tag{2}$$

where $J_1 = J_2$ and $h \equiv h_2$. The eigenvalues of this matrix read

$$\lambda_1 = \frac{1}{2} J_1 \Delta - \sqrt{2J_1^2 + \left(h + \frac{1}{2} J_1 \Delta\right)^2}, \tag{3a}$$

$$\lambda_2 = -h, \tag{3b}$$

$$\lambda_3 = \frac{1}{2} J_1 \Delta + \sqrt{2J_1^2 + \left(h + \frac{1}{2} J_1 \Delta\right)^2}, \tag{3c}$$

and the corresponding non-normalized eigenvectors are

$$|\Psi_1\rangle = \{1, -\frac{h + \lambda_1}{J_1}, 1\}, \tag{4a}$$

$$|\Psi_2\rangle = \{1, 0, -1\}, \tag{4b}$$

$$|\Psi_3\rangle = \{1, -\frac{h + \lambda_3}{J_1}, 1\}. \tag{4c}$$

To proceed further, we expand the initial and final states in the basis of eigenvectors from Supplementary Equation (4), i.e.,

$$|\uparrow\downarrow\downarrow\rangle = \sum_{k=1}^{N} a_k^{(1)} |\Psi_k\rangle, \tag{5a}$$

$$|\downarrow\downarrow\uparrow\rangle = \sum_{k=1}^{N} a_k^{(3)} |\Psi_k\rangle, \tag{5b}$$

where $a_k^{(i)}$ are the expansion coefficients, the index $i = 1, 2, 3$ denotes the expanded spin state. Note that since the Hamiltonian is a bisymmetric matrix, the coefficients $a_k^{(1)}$ and $a_k^{(3)}$ are related as[1,2] $a_k^{(1)} = (-1)^{k+1} a_k^{(3)}$. Therefore, if we apply the evolution operator $U(t) = e^{-iHt}$ to the initial state, we obtain the condition for perfect state transfer at t_{out},

$U(t_{\text{out}})\,|{\uparrow\downarrow\downarrow}\rangle = |{\downarrow\downarrow\uparrow}\rangle$, in the following form

$$\sum_{k=1}^{N}[e^{-i\lambda_k t_{\text{out}}} - (-1)^{k+1}]a_k^{(1)}\,|\Psi_k\rangle = 0. \tag{6}$$

This condition can be rewritten as

$$(\lambda_2 - \lambda_1)t_{\text{out}} = (2m_1 + 1)\pi, \tag{7a}$$

$$(\lambda_3 - \lambda_2)t_{\text{out}} = (2m_2 - 1)\pi, \tag{7b}$$

where m_1 and m_2 are non-negative integers, moreover $m_2 > 0$, as we assume that $\lambda_1 < \lambda_2 < \lambda_3$. From these conditions, we can determine the magnetic field h for the transfer to occur during the shortest possible time, $\min t_{\text{out}} \equiv t_{\text{min}}$. We obtain $m_1 = 0$, $m_2 = 1$ and $\frac{\lambda_2-\lambda_1}{\lambda_3-\lambda_2} = 1$. The corresponding magnetic field is

$$h = -\frac{1}{2}J_1\Delta. \tag{8}$$

The minimal time interval for transfer is $t_{\text{min}} = \frac{\pi}{\lambda_2-\lambda_1} = \frac{\pi}{\sqrt{2}|J_1|}$. Note that the energies of the initial $|{\uparrow\downarrow\downarrow}\rangle$ and final $|{\downarrow\downarrow\uparrow}\rangle$ states, $\frac{1}{2}J_1\Delta$, are equal to the energy of the intermediate state $|{\downarrow\uparrow\downarrow}\rangle$, so perfect transfer for a three-level system is achieved when the intermediate state is resonant with the initial and final states. Upon the transfer of a single spin excitation in a three-state system, the amplitude of the final state $|{\downarrow\downarrow\uparrow}\rangle$ is $\big(-i\,\text{sgn}(J_1)\big)\big(-i\,\text{sgn}(J_2)\big) = -1$, with $J_1 = J_2$, which means that the final state acquires the phase $\phi = \pi$ (or sign change) relative to the initial state $|{\uparrow\downarrow\downarrow}\rangle$. These are of course obvious results, but their derivation can be useful for examining more complicated cases with $N > 3$.

Consider now the spin chain with two excitations. In the basis of $\{|{\uparrow\uparrow\downarrow}\rangle, |{\uparrow\downarrow\uparrow}\rangle, |{\downarrow\uparrow\uparrow}\rangle\}$, the Hamiltonian matrix reads

$$H = \begin{pmatrix} h & -J_1 & 0 \\ -J_1 & -h + J_1\Delta & -J_1 \\ 0 & -J_1 & h \end{pmatrix}, \tag{9}$$

Our goal is that the initial state $|{\uparrow\uparrow\downarrow}\rangle$ does not evolve in time, i.e., the control spin at the $j = 2$ site does not leak out to the site $j = 3$ while blocking the transfer of spin excitation from the site $j = 1$. We thus require that the initial state $|{\uparrow\uparrow\downarrow}\rangle$ (and state $|{\downarrow\uparrow\uparrow}\rangle$) be out of resonance with state $|{\uparrow\downarrow\uparrow}\rangle$. This leads to the condition $|J_1| \ll |J_1\Delta - 2h|$, which, for the value of the magnetic field in Supplementary Equation (8), reduces to $|2\Delta| \gg 1$. This is the Ising limit of our spin-chain Hamiltonian, which is a rather trivial and impractical case, as it would also require a large magnetic field $|h| \gg |J_1|$.

Note finally that in order to use the spin transistor for various quantum information tasks described in the main text, the spin excitation on the gate site(s) should not leak out to the output or input ports, even when alone. Obviously, in the spin

chain with $N = 3$ and the magnetic field as in Supplementary Equation (8), such an initial state $|\downarrow\uparrow\downarrow\rangle$ resonantly couples to the states $|\uparrow\downarrow\downarrow\rangle$ and $|\downarrow\downarrow\uparrow\rangle$, and therefore the three-site system is not suitable for our purposes. In Supplementary Notes 2 and 3 we show that spin chains with $N \geq 4$ can be used to engineer a quantum spin transistor for any value of Δ, except for the very special case of $\Delta = 0$ corresponding to the XX model.

Supplementary Note 2: $N = 4$ spin chain.

Here we provide details of calculations for the values of gate magnetic field h_j required for the realization of quantum spin transistor in a Heisenberg XXZ spin chain described by Supplementary Equation (1) with $N = 4$, $h_{1,N} = 0$ and $J_i = J_{N-i}$. We first consider the $N_\uparrow = 1$ and $N_\downarrow = 3$ spin chain, which has two different interaction coefficients, $J_1(= J_3)$ and J_2, and we assume that $h_{2,3} = h$. For simplicity, we focus our discussion on a chain with $J_i > 0$. However, we have checked that the derived conclusions also hold true for $J_i < 0$.

In the basis of $\{|\uparrow\downarrow\downarrow\downarrow\rangle, |\downarrow\uparrow\downarrow\downarrow\rangle, |\downarrow\downarrow\uparrow\downarrow\rangle, |\downarrow\downarrow\downarrow\uparrow\rangle\}$, the Hamiltonian matrix is

$$H = \begin{pmatrix} -2h - \frac{1}{2}J_2\Delta & -J_1 & 0 & 0 \\ -J_1 & \frac{1}{2}J_2\Delta & -J_2 & 0 \\ 0 & -J_2 & \frac{1}{2}J_2\Delta & -J_1 \\ 0 & 0 & -J_1 & -2h - \frac{1}{2}J_2\Delta \end{pmatrix}. \tag{10}$$

We assume that the strongest interaction is between the states $|\downarrow\uparrow\downarrow\downarrow\rangle$ and $|\downarrow\downarrow\uparrow\downarrow\rangle$, i.e., $J_2 \gg J_1$. To utilize this fact, we prediagonalize the Hamiltonian. To this end, we first find the eigenvalues and eigenvectors of the inner block (gate) $\begin{pmatrix} \frac{1}{2}J_2\Delta & -J_2 \\ -J_2 & \frac{1}{2}J_2\Delta \end{pmatrix}$, they are $\lambda_+ = \frac{1}{2}J_2(\Delta - 2)$, $\lambda_- = \frac{1}{2}J_2(\Delta + 2)$, and $|G_+\rangle = \frac{1}{\sqrt{2}}(|\uparrow\downarrow\rangle + |\downarrow\uparrow\rangle)$, $|G_-\rangle = \frac{1}{\sqrt{2}}(|\uparrow\downarrow\rangle - |\downarrow\uparrow\rangle)$. Then we introduce a new basis $\left\{|\uparrow\downarrow\downarrow\downarrow\rangle, \frac{1}{\sqrt{2}}(|\downarrow\uparrow\downarrow\downarrow\rangle + |\downarrow\downarrow\uparrow\downarrow\rangle), \frac{1}{\sqrt{2}}(|\downarrow\uparrow\downarrow\downarrow\rangle - |\downarrow\downarrow\uparrow\downarrow\rangle), |\downarrow\downarrow\downarrow\uparrow\rangle\right\}$, and the corresponding unitary transformation

$$O = \begin{pmatrix} 1 & 0 & 0 & 0 \\ 0 & \frac{1}{\sqrt{2}} & \frac{1}{\sqrt{2}} & 0 \\ 0 & \frac{1}{\sqrt{2}} & -\frac{1}{\sqrt{2}} & 0 \\ 0 & 0 & 0 & 1 \end{pmatrix}, \tag{11}$$

which, when applied to the Hamiltonian in Supplementary Equation (10) leads to

$$\tilde{H} = \begin{pmatrix} -2h - \frac{1}{2}J_2\Delta & -\frac{1}{\sqrt{2}}J_1 & -\frac{1}{\sqrt{2}}J_1 & 0 \\ -\frac{1}{\sqrt{2}}J_1 & \frac{1}{2}J_2\Delta - J_2 & 0 & -\frac{1}{\sqrt{2}}J_1 \\ -\frac{1}{\sqrt{2}}J_1 & 0 & \frac{1}{2}J_2\Delta + J_2 & \frac{1}{\sqrt{2}}J_1 \\ 0 & -\frac{1}{\sqrt{2}}J_1 & \frac{1}{\sqrt{2}}J_1 & -2h - \frac{1}{2}J_2\Delta \end{pmatrix}. \tag{12}$$

Note that in the limit of $J_1 \to 0$ this Hamiltonian is diagonal, with the energy levels

$$\tilde{\lambda}_1 = -2h - \frac{1}{2}J_2\Delta, \tag{13a}$$

$$\tilde{\lambda}_2 = \frac{1}{2}J_2(\Delta - 2), \tag{13b}$$

$$\tilde{\lambda}_3 = \frac{1}{2}J_2(\Delta + 2), \tag{13c}$$

$$\tilde{\lambda}_4 = -2h - \frac{1}{2}J_2\Delta. \tag{13d}$$

We can now find magnetic fields h needed to achieve perfect spin transfer. Spin excitation transfer can be obtained if there are three levels resonant with each other, i.e., either $\tilde{\lambda}_{1,4} = \tilde{\lambda}_2 (= \lambda_+)$ or $\tilde{\lambda}_{1,4} = \tilde{\lambda}_3 (= \lambda_-)$. The two values of the magnetic field that satisfy these conditions are

$$h_+ = \frac{1}{2}J_2(1 - \Delta), \tag{14a}$$

$$h_- = -\frac{1}{2}J_2(1 + \Delta). \tag{14b}$$

Hence, for any Δ it is possible to reduce this spin chain to an effective three-state system.

To verify that (nearly) perfect transfer is indeed achieved, we use the same approach as for the three-spin case. The eigenvalues of the Hamiltonian in Supplementary Equation (10) are

$$\lambda_1 = -\frac{1}{2}J_2 - h - \sqrt{J_1^2 + \left(h - \frac{1}{2}J_2(1 - \Delta)\right)^2}, \tag{15a}$$

$$\lambda_2 = \frac{1}{2}J_2 - h - \sqrt{J_1^2 + \left(h + \frac{1}{2}J_2(1 + \Delta)\right)^2}, \tag{15b}$$

$$\lambda_3 = -\frac{1}{2}J_2 - h + \sqrt{J_1^2 + \left(h - \frac{1}{2}J_2(1 - \Delta)\right)^2}, \tag{15c}$$

$$\lambda_4 = \frac{1}{2}J_2 - h + \sqrt{J_1^2 + \left(h + \frac{1}{2}J_2(1 + \Delta)\right)^2}, \tag{15d}$$

and the corresponding non-normalized eigenvectors in the basis of $\{|\uparrow\downarrow\downarrow\downarrow\rangle, |\downarrow\uparrow\downarrow\downarrow\rangle, |\downarrow\downarrow\uparrow\downarrow\rangle, |\downarrow\downarrow\downarrow\uparrow\rangle\}$ are

$$|\Psi_1\rangle = \left\{1, \frac{J_1^2 + J_2((1-\frac{1}{2}\Delta)J_2 + \lambda_3)}{J_1\left(\frac{1}{2}J_2\Delta - \lambda_1\right)}, \frac{J_2(1-\frac{1}{2}\Delta)+\lambda_3}{J_1}, 1\right\}, \tag{16a}$$

$$|\Psi_2\rangle = \left\{1, \frac{J_1^2 + J_2((1+\frac{1}{2}\Delta)J_2 - \lambda_4)}{J_1(\frac{1}{2}J_2\Delta - \lambda_2)}, \frac{J_2(1+\frac{1}{2}\Delta)-\lambda_4}{J_1}, -1\right\}, \tag{16b}$$

$$|\Psi_3\rangle = \left\{1, \frac{J_1^2 + J_2\left((1-\frac{1}{2}\Delta)J_2 + \lambda_1\right)}{J_1\left(\frac{1}{2}J_2\Delta - \lambda_3\right)}, \frac{J_2(1-\frac{1}{2}\Delta)+\lambda_1}{J_1}, 1\right\}, \tag{16c}$$

$$|\Psi_4\rangle = \left\{1, \frac{J_1^2 + J_2\left((1+\frac{1}{2}\Delta)J_2 - \lambda_2\right)}{J_1\left(\frac{1}{2}J_2\Delta - \lambda_4\right)}, \frac{J_2(1+\frac{1}{2}\Delta)-\lambda_2}{J_1}, -1\right\}. \tag{16d}$$

As before, we expand the initial and final states in the basis of eigenvectors from Supplementary Equation (16),

$$|\uparrow\downarrow\downarrow\downarrow\rangle = \sum_{k=1}^{N} a_k^{(1)}\,|\Psi_k\rangle, \tag{17a}$$

$$|\downarrow\downarrow\downarrow\uparrow\rangle = \sum_{k=1}^{N} a_k^{(4)}\,|\Psi_k\rangle. \tag{17b}$$

Since the Hamiltonian in Supplementary Equation (10) is a bisymmetric matrix, the expansion coefficients $a_k^{(i)}$ are related as[1,2] $a_1^{(1)} = a_1^{(4)}$, $a_2^{(1)} = -a_2^{(4)}$, $a_3^{(1)} = a_3^{(4)}$, $a_4^{(1)} = -a_4^{(4)}$. Hence, the necessary and sufficient conditions for the state $|\uparrow\downarrow\downarrow\downarrow\rangle$ to evolve into the state $|\downarrow\downarrow\downarrow\uparrow\rangle$ during time t_{out} are

$$(\lambda_{k+1} - \lambda_k)t_{\text{out}} = \pi(2m_k + 1), \tag{18}$$

where m_k is a positive integer.

We now use the values of the magnetic field from Supplementary Equation (14) to determine the fastest transfer time, $t_{\min} = \frac{\pi}{\delta\lambda}$, where $\delta\lambda$ is the energy difference between the equidistant levels. Firstly, we take h_+, for which we obtain

$$\lambda_2 - \lambda_1 = J_1 + J_2 - \sqrt{J_1^2 + J_2^2} \approx J_1, \tag{19a}$$

$$\lambda_3 - \lambda_2 = J_1 - J_2 + \sqrt{J_1^2 + J_2^2} \approx J_1, \tag{19b}$$

$$\lambda_4 - \lambda_3 = -J_1 + J_2 + \sqrt{J_1^2 + J_2^2} \approx -J_1 + 2J_2. \tag{19c}$$

We see that for $J_1/J_2 \ll 1$ the lowest three energy levels of the system are equidistant, with the difference $\delta\lambda = J_1$, and the highest energy level lies far away from the others. That is why we can expect nearly perfect transfer at time $t_{\min} = \frac{\pi}{J_1}$. Secondly, we take h_-, for which we have

$$\lambda_2 - \lambda_1 = J_2 - J_1 + \sqrt{J_1^2 + J_2^2} \approx 2J_2 - J_1 \tag{20a}$$

$$\lambda_3 - \lambda_2 = J_1 - J_2 + \sqrt{J_1^2 + J_2^2} \approx J_1 \tag{20b}$$

$$\lambda_4 - \lambda_3 = J_1 + J_2 - \sqrt{J_1^2 + J_2^2} \approx J_1 \tag{20c}$$

with the higher three levels equidistant and the lower level is much further below the other three.

Let us now illustrate our findings using the particular values of $\Delta = -1$ and $h_- = -\frac{1}{2}J_2(1+\Delta) = 0$. The corresponding normalized eigenstates are obtained from Supplementary Equation (16)

$$|\Psi_1\rangle = \frac{1}{2\sqrt{J_1^2+J_2^2+J_2\sqrt{J_1^2+J_2^2}}}\left\{J_1, J_2+\sqrt{J_1^2+J_2^2}, J_2+\sqrt{J_1^2+J_2^2}, J_1\right\}, \tag{21a}$$

$$|\Psi_2\rangle = \frac{1}{2}\{1,1,-1,-1\}, \tag{21b}$$

$$|\Psi_3\rangle = \frac{1}{2\sqrt{J_1^2+J_2^2-J_2\sqrt{J_1^2+J_2^2}}}\left\{J_1, J_2-\sqrt{J_1^2+J_2^2}, J_2-\sqrt{J_1^2+J_2^2}, J_1\right\}, \tag{21c}$$

$$|\Psi_4\rangle = \frac{1}{2}\{1,-1,1,-1\}, \tag{21d}$$

and the spin states expanded in this basis are

$$|{\uparrow\downarrow\downarrow\downarrow}\rangle = \frac{1}{2}\left\{\frac{J_1}{\sqrt{J_1^2+J_2^2+J_2\sqrt{J_1^2+J_2^2}}}, 1, \frac{J_1}{\sqrt{J_1^2+J_2^2-J_2\sqrt{J_1^2+J_2^2}}}, 1\right\}_\Psi, \tag{22a}$$

$$|{\downarrow\uparrow\downarrow\downarrow}\rangle = \frac{1}{2}\left\{\frac{J_2+\sqrt{J_1^2+J_2^2}}{\sqrt{J_1^2+J_2^2+J_2\sqrt{J_1^2+J_2^2}}}, 1, \frac{J_2-\sqrt{J_1^2+J_2^2}}{\sqrt{J_1^2+J_2^2-J_2\sqrt{J_1^2+J_2^2}}}, -1\right\}_\Psi, \tag{22b}$$

$$|{\downarrow\downarrow\uparrow\downarrow}\rangle = \frac{1}{2}\left\{\frac{J_2+\sqrt{J_1^2+J_2^2}}{\sqrt{J_1^2+J_2^2+J_2\sqrt{J_1^2+J_2^2}}}, -1, \frac{J_2-\sqrt{J_1^2+J_2^2}}{\sqrt{J_1^2+J_2^2-J_2\sqrt{J_1^2+J_2^2}}}, 1\right\}_\Psi, \tag{22c}$$

$$|{\downarrow\downarrow\downarrow\uparrow}\rangle = \frac{1}{2}\left\{\frac{J_1}{\sqrt{J_1^2+J_2^2+J_2\sqrt{J_1^2+J_2^2}}}, -1, \frac{J_1}{\sqrt{J_1^2+J_2^2-J_2\sqrt{J_1^2+J_2^2}}}, -1\right\}_\Psi. \tag{22d}$$

In the limit of $J_1/J_2 \ll 1$, these expressions reduce to

$$|{\uparrow\downarrow\downarrow\downarrow}\rangle \simeq \frac{1}{2}\left\{\frac{J_1}{\sqrt{2}J_2}, 1, \sqrt{2}, 1\right\}_\Psi, \tag{23a}$$

$$|{\downarrow\uparrow\downarrow\downarrow}\rangle \simeq \frac{1}{2}\left\{\sqrt{2}, 1, -\frac{J_1}{\sqrt{2}J_2}, -1\right\}_\Psi, \tag{23b}$$

$$|{\downarrow\downarrow\uparrow\downarrow}\rangle \simeq \frac{1}{2}\left\{\sqrt{2}, -1, -\frac{J_1}{\sqrt{2}J_2}, 1\right\}_\Psi, \tag{23c}$$

$$|{\downarrow\downarrow\downarrow\uparrow}\rangle \simeq \frac{1}{2}\left\{\frac{J_1}{\sqrt{2}J_2}, -1, \sqrt{2}, -1\right\}_\Psi. \tag{23d}$$

We see that for all spin states one of the coefficients of the expansion in the basis of energy eigenstates is much smaller than the other three. Hence, we indeed have an effective three-level system for spin transfer between the input $j = 1$ and output $j = 4$ ports via the intermediate resonant $|{\downarrow}\, G_-\, {\downarrow}\rangle$ state. In the main text, we use the following notation for such input and output states:

$$|{\uparrow\downarrow\downarrow\downarrow}\rangle \equiv |{\uparrow}\rangle_{\text{in}}\,|0\rangle_{\text{gate}}\,|{\downarrow}\rangle_{\text{out}},$$

$$|{\downarrow\downarrow\downarrow\uparrow}\rangle \equiv |{\downarrow}\rangle_{\text{in}}\,|0\rangle_{\text{gate}}\,|{\uparrow}\rangle_{\text{out}}.$$

Note that if we place a spin excitation in the non-resonant $|G_+\rangle$ state of the gate (sites 2 and 3), it will stay there indefinitely (assuming no energy relaxations) due to the large energy mismatch $\sim 2J_2$ to the other states; such a state in our notation would be

$$|\downarrow G_+ \downarrow\rangle \equiv |\downarrow\rangle_{\rm in} |1\rangle_{\rm gate} |\downarrow\rangle_{\rm out} .$$

Recall that we set the gate magnetic field to h_-. If, instead, we chose h_+ then the roles of the $|G_-\rangle$ and $|G_+\rangle$ states of the gate would be interchanged, i.e., $|\downarrow G_+ \downarrow\rangle$ would be the intermediate resonant state for the spin transfer between $|\uparrow\rangle_{\rm in} |0\rangle_{\rm gate} |\downarrow\rangle_{\rm out}$ and $|\downarrow\rangle_{\rm in} |0\rangle_{\rm gate} |\uparrow\rangle_{\rm out}$, while $|\downarrow G_- \downarrow\rangle \equiv |\downarrow\rangle_{\rm in} |1\rangle_{\rm gate} |\downarrow\rangle_{\rm out}$ would be the trapped (non-evolving) state. The only difference now is that, upon the spin transfer, the output state will acquire the phase shift $\phi = \pi$ (sign change) relative to the input state, since the symmetric resonant intermediate state couples to the input and output states with the same constant $-\frac{1}{\sqrt{2}}J_1$, see Supplementary Equation (12).

Using the derivations above, it is straightforward to analyze chains with $J_i < 0$. The only difference is that in this case the energies λ_i in Supplementary Equation (15) should be reordered to form an increasing sequence. When this is done, we take the same steps as before and conclude that for $J_i < 0$ it is also always possible to construct a three-level system. To illustrate this case, we consider the example from the main text with $h_- = 0$, for which we obtain $\lambda_2(\simeq J_2/2 - |J_1|) < \lambda_1(\simeq J_2/2) < \lambda_4(\simeq J_2/2 + |J_1|) < \lambda_3(\simeq -J_2/2)$. We see that the lowest three energy levels are equidistant leading to perfect state transfer at $t_{min} = \pi/|J_1|$. Note also that from the Hamiltonian in Supplementary Equation (12), we have that the coupling constants $\mp\frac{1}{\sqrt{2}}J_1$ of the intermediate antisymmetric state $|\downarrow G_- \downarrow\rangle$ to the input and output states have opposite sign. This means that upon the transfer of the single spin excitation from the input to the output ports, the amplitude of the final state $(-i)(i) = 1$ is independent of the sign of J_1.

Consider now the two-excitation case, $N_\uparrow = 2$, $N_\downarrow = 2$. In the basis of $\{|\uparrow\uparrow\downarrow\downarrow\rangle, |\uparrow\downarrow\uparrow\downarrow\rangle, |\uparrow\downarrow\downarrow\uparrow\rangle, |\downarrow\uparrow\uparrow\downarrow\rangle, |\downarrow\uparrow\downarrow\uparrow\rangle, |\downarrow\downarrow\uparrow\uparrow\rangle\}$, the Hamiltonian H is

$$\begin{pmatrix} (-J_1+\frac{1}{2}J_2)\Delta & -J_2 & 0 & 0 & 0 & 0 \\ -J_2 & (J_1+\frac{1}{2}J_2)\Delta & -J_1 & -J_1 & 0 & 0 \\ 0 & -J_1 & (J_1-\frac{1}{2}J_2)\Delta - 2h & 0 & -J_1 & 0 \\ 0 & -J_1 & 0 & (J_1-\frac{1}{2}J_2)\Delta + 2h & -J_1 & 0 \\ 0 & 0 & -J_1 & -J_1 & (J_1+\frac{1}{2}J_2)\Delta & -J_2 \\ 0 & 0 & 0 & 0 & -J_2 & (-J_1+\frac{1}{2}J_2)\Delta \end{pmatrix}, \quad (24)$$

By construction, the exchange interaction J_2 between the gate sites $j = 2-3$ is the strongest. We again prediagonalize the Hamiltonian and change the basis to

$$\{|\uparrow G_+ \downarrow\rangle, |\uparrow G_- \downarrow\rangle, |\uparrow\downarrow\downarrow\uparrow\rangle, |\downarrow\uparrow\uparrow\downarrow\rangle, |\downarrow G_+ \uparrow\rangle, |\downarrow G_- \uparrow\rangle\}$$

with $|G_+\rangle \equiv \frac{1}{\sqrt{2}}(|\uparrow\downarrow\rangle + |\downarrow\uparrow\rangle)$ and $|G_-\rangle \equiv \frac{1}{\sqrt{2}}(|\uparrow\downarrow\rangle - |\downarrow\uparrow\rangle)$ being the two eigenstates of the gate part of the chain. Using the unitary transformation

$$O = \begin{pmatrix} \frac{1}{\sqrt{2}} & \frac{1}{\sqrt{2}} & 0 & 0 & 0 & 0 \\ \frac{1}{\sqrt{2}} & -\frac{1}{\sqrt{2}} & 0 & 0 & 0 & 0 \\ 0 & 0 & 1 & 0 & 0 & 0 \\ 0 & 0 & 0 & 1 & 0 & 0 \\ 0 & 0 & 0 & 0 & \frac{1}{\sqrt{2}} & \frac{1}{\sqrt{2}} \\ 0 & 0 & 0 & 0 & \frac{1}{\sqrt{2}} & -\frac{1}{\sqrt{2}} \end{pmatrix}, \tag{25}$$

we transform the Hamiltonian in Supplementary Equation (24) into

$$\tilde{H} = \begin{pmatrix} -J_2(1-\frac{1}{2}\Delta) & -J_1\Delta & -\frac{1}{\sqrt{2}}J_1 & -\frac{1}{\sqrt{2}}J_1 & 0 & 0 \\ -J_1\Delta & J_2(1+\frac{1}{2}\Delta) & \frac{1}{\sqrt{2}}J_1 & \frac{1}{\sqrt{2}}J_1 & 0 & 0 \\ -\frac{1}{\sqrt{2}}J_1 & \frac{1}{\sqrt{2}}J_1 & (J_1-\frac{1}{2}J_2)\Delta - 2h & 0 & -\frac{1}{\sqrt{2}}J_1 & -\frac{1}{\sqrt{2}}J_1 \\ -\frac{1}{\sqrt{2}}J_1 & \frac{1}{\sqrt{2}}J_1 & 0 & (J_1-\frac{1}{2}J_2)\Delta + 2h & -\frac{1}{\sqrt{2}}J_1 & -\frac{1}{\sqrt{2}}J_1 \\ 0 & 0 & -\frac{1}{\sqrt{2}}J_1 & -\frac{1}{\sqrt{2}}J_1 & -J_2(1-\frac{1}{2}\Delta) & J_1\Delta \\ 0 & 0 & -\frac{1}{\sqrt{2}}J_1 & -\frac{1}{\sqrt{2}}J_1 & J_1\Delta & J_2(1+\frac{1}{2}\Delta) \end{pmatrix}. \tag{26}$$

We place one (control) spin-up in the gate, either in the state $|G_+\rangle = |1\rangle_{\text{gate}}$, if we set the gate magnetic field to h_-, or in the state $|G_-\rangle = |1\rangle_{\text{gate}}$, if we set it to h_+. As stated above, the control spin-up then cannot leak out of the gate, i.e., $|\downarrow\rangle_{\text{in}} |1\rangle_{\text{gate}} |\downarrow\rangle_{\text{out}}$ is stationary. Next, we place the target spin-up on the site $j = 1$, obtaining the state $|\uparrow\rangle_{\text{in}} |1\rangle_{\text{gate}} |\downarrow\rangle_{\text{out}}$. We now verify that this state does not evolve since it is non-resonant with all the other states to which it can couple with rates $\sim J_1 \ll J_2$.

Assuming $h_- = -\frac{1}{2}J_2(1+\Delta)$, the energy of the initial state $|\uparrow G_+ \downarrow\rangle$ is

$$\tilde{\lambda}_{\uparrow G_+ \downarrow} = -J_2\left(1 - \frac{1}{2}\Delta\right), \tag{27}$$

while the energies of states $|\uparrow\downarrow\downarrow\uparrow\rangle$ and $|\downarrow\uparrow\uparrow\downarrow\rangle$ are

$$\tilde{\lambda}_{\uparrow\downarrow\downarrow\uparrow} \simeq -\frac{1}{2}J_2\Delta - 2h_- = J_2\left(1+\frac{1}{2}\Delta\right), \tag{28a}$$

$$\tilde{\lambda}_{\downarrow\uparrow\uparrow\downarrow} \simeq -\frac{1}{2}J_2\Delta + 2h_- = -J_2\left(1+\frac{3}{2}\Delta\right). \tag{28b}$$

Unless $\Delta = 0$, the initial state $|\uparrow G_+ \downarrow\rangle$ is highly non-resonant with all the other connected states. State $|\downarrow G_+ \uparrow\rangle$ has of course the same energy as $|\uparrow G_+ \downarrow\rangle$, but these states are not directly connected via the first-order spin-exchange interaction. The second order couplings between these states via the non-resonant intermediate states $|\downarrow\uparrow\uparrow\downarrow\rangle$ and $|\uparrow\downarrow\downarrow\uparrow\rangle$ detuned by $\delta\tilde{\lambda}_a = 2\Delta J_2$ and $\delta\tilde{\lambda}_b = -2J_2$ have the amplitudes $J_a^{(2)} = (J_1/\sqrt{2})^2/(-2\Delta J_2)$ and $J_b^{(2)} = (J_1/\sqrt{2})^2/(2J_2)$, respectively, which are both small compared to $J_1 \ll J_2$. During the transfer time $t_{\text{out}} = \pi/J_1$, the probability of transition $|\uparrow G_+ \downarrow\rangle \to |\downarrow G_+ \uparrow\rangle$ is then $(2J^{(2)}t_{\text{out}})^2 = (\pi/2)^2(J_1/J_2)^2$, where we took $\Delta = -1$ and the factor of 2 comes from the two transition paths having the same amplitude $J_a^{(2)} = J_b^{(2)} = J^{(2)}$. Hence, this transition probability is highly suppressed and can therefore be neglected, which is verified by our exact numerical simulations [see Fig. 1c of the main text].

Similarly, for $h_+ = \frac{1}{2}J_2(1-\Delta)$, the energy of the initial state $|\uparrow G_- \downarrow\rangle$ is

$$\tilde{\lambda}_{\uparrow G_-\downarrow} = J_2\left(1+\frac{1}{2}\Delta\right), \tag{29}$$

while the energies of states $|\uparrow\downarrow\downarrow\uparrow\rangle$ and $|\downarrow\uparrow\uparrow\downarrow\rangle$ are

$$\tilde{\lambda}_{\uparrow\downarrow\downarrow\uparrow} \simeq -\frac{1}{2}J_2\Delta - 2h_+ = -J_2\left(1-\frac{1}{2}\Delta\right), \tag{30a}$$

$$\tilde{\lambda}_{\downarrow\uparrow\uparrow\downarrow} \simeq -\frac{1}{2}J_2\Delta + 2h_+ = J_2\left(1-\frac{3}{2}\Delta\right), \tag{30b}$$

and again we have a non-resonant initial state $|\uparrow G_- \downarrow\rangle$ which it is not directly connected to $|\downarrow G_- \uparrow\rangle$.

Supplementary Note 3: $N = 5$ spin chain.

Here we provide details of calculations for the values of gate magnetic field h_j required for the realization of quantum spin transistor in the Heisenberg XXZ spin chain described by Supplementary Equation (1) with $N = 5$, $h_{1,N} = 0$ and $J_i = J_{N-i}$. The five-spin chain does not differ much, in principle, from the four-spin chain. So in the spirit of Supplementary Note 2, we first determine the values of magnetic field for which spin excitation transfer is achieved in the $N_\uparrow = 1$, $N_\downarrow = 4$ spin chain, characterized by the two interaction coefficients J_1 and J_2, with the condition $J_1/J_2 \ll 1$. However, the magnetic field in the middle of the chain might not be the same on different sites, and we therefore assume: $h_2 = h_4 = h'$ and $h_3 = h$.

In the basis of $\{|\uparrow\downarrow\downarrow\downarrow\downarrow\rangle, |\downarrow\uparrow\downarrow\downarrow\downarrow\rangle, |\downarrow\downarrow\uparrow\downarrow\downarrow\rangle, |\downarrow\downarrow\downarrow\uparrow\downarrow\rangle, |\downarrow\downarrow\downarrow\downarrow\uparrow\rangle\}$, the Hamiltonian for this system is

$$H = \begin{pmatrix} -2h' - h - J_2\Delta & -J_1 & 0 & 0 & 0 \\ -J_1 & -h & -J_2 & 0 & 0 \\ 0 & -J_2 & h - 2h' + (J_2 - J_1)\Delta & -J_2 & 0 \\ 0 & 0 & -J_2 & -h & -J_1 \\ 0 & 0 & 0 & -J_1 & -2h' - h - J_2\Delta \end{pmatrix}. \tag{31}$$

By assumption, the exchange interaction is the strongest between the states $|\downarrow\uparrow\downarrow\downarrow\downarrow\rangle, |\downarrow\downarrow\uparrow\downarrow\downarrow\rangle, |\downarrow\downarrow\downarrow\uparrow\downarrow\rangle$, so we once again prediagonalize the Hamiltonian by finding the eigenvalues and eigenvectors of the gate matrix

$$\begin{pmatrix} -h & -J_2 & 0 \\ -J_2 & h - 2h' + (J_2 - J_1)\Delta & -J_2 \\ 0 & -J_2 & -h \end{pmatrix}. \tag{32}$$

We write the eigenvalues as

$$\lambda_+ = -h' + \frac{1}{2}(J_2 - J_1)\Delta + \sqrt{2J_2^2 + \left(h - h' + \frac{1}{2}(J_2 - J_1)\Delta\right)^2}, \tag{33a}$$

$$\lambda_0 = -h, \tag{33b}$$

$$\lambda_- = -h' + \frac{1}{2}(J_2 - J_1)\Delta - \sqrt{2J_2^2 + \left(h - h' + \frac{1}{2}(J_2 - J_1)\Delta\right)^2}, \tag{33c}$$

and the corresponding normalized eigenvectors in the basis of $\{|\uparrow\downarrow\downarrow\rangle, |\downarrow\uparrow\downarrow\rangle, |\downarrow\downarrow\uparrow\rangle\}$ are

$$|G_+\rangle = \frac{1}{\sqrt{2J_2^2 + (h + \lambda_+)^2}}\{J_2, -(h + \lambda_+), J_2\}, \tag{34a}$$

$$|G_0\rangle = \frac{1}{\sqrt{2}}\{1, 0, -1\}, \tag{34b}$$

$$|G_-\rangle = \frac{1}{\sqrt{2J_2^2 + (h + \lambda_-)^2}}\{J_2, -(h + \lambda_-), J_2\}. \tag{34c}$$

We introduce a new basis $\{|\uparrow\downarrow\downarrow\downarrow\downarrow\rangle, |\downarrow G_+ \downarrow\rangle, |\downarrow G_0 \downarrow\rangle, |\downarrow G_- \downarrow\rangle, |\downarrow\downarrow\downarrow\downarrow\uparrow\rangle\}$ and the corresponding unitary transformation

$$U = \begin{pmatrix} 1 & 0 & 0 & 0 & 0 \\ 0 & \frac{J_2}{\sqrt{2J_2^2+(h+\lambda_+)^2}} & -\frac{h+\lambda_+}{\sqrt{2J_2^2+(h+\lambda_+)^2}} & \frac{J_2}{\sqrt{2J_2^2+(h+\lambda_+)^2}} & 0 \\ 0 & \frac{1}{\sqrt{2}} & 0 & -\frac{1}{\sqrt{2}} & 0 \\ 0 & \frac{J_2}{\sqrt{2J_2^2+(h+\lambda_-)^2}} & -\frac{h+\lambda_-}{\sqrt{2J_2^2+(h+\lambda_-)^2}} & \frac{J_2}{\sqrt{2J_2^2+(h+\lambda_-)^2}} & 0 \\ 0 & 0 & 0 & 0 & 1 \end{pmatrix}. \tag{35}$$

As before, the transformed Hamiltonian is nearly diagonal when $J_1/J_2 \ll 1$,

$$\tilde{H} \simeq \begin{pmatrix} -2h' - h - J_2\Delta & 0 & 0 & 0 & 0 \\ 0 & \lambda_+ & 0 & 0 & 0 \\ 0 & 0 & \lambda_0 & 0 & 0 \\ 0 & 0 & 0 & \lambda_- & 0 \\ 0 & 0 & 0 & 0 & -2h' - h - J_2\Delta \end{pmatrix}. \quad (36)$$

To achieve resonant transfer between states $|\uparrow\downarrow\downarrow\downarrow\downarrow\rangle$ and $|\downarrow\downarrow\downarrow\downarrow\uparrow\rangle$, we need to find the values of magnetic field so that one of λ_+, λ_- or λ_0 is equal to $-2h' - h - J_2\Delta$. For instance, from the condition $-2h' - h - J_2\Delta = \lambda_0$ we find $h' = -\frac{1}{2}J_2\Delta$ and any h. In this case, the spin transfer goes via the resonant intermediate state $|\downarrow G_0 \downarrow\rangle$. The condition $-2h' - h - J_2\Delta = \lambda_+$ can be satisfied if

$$h' = \frac{J_2^2}{2(h + J_2\Delta)} - \frac{1}{2}J_2\Delta \ \ \& \ \ h < -J_2\Delta. \quad (37)$$

Alternatively, the condition $-2h' - h - J_2\Delta = \lambda_-$ gives

$$h' = \frac{J_2^2}{2(h + J_2\Delta)} - \frac{1}{2}J_2\Delta \ \ \& \ \ h > -J_2\Delta. \quad (38)$$

Therefore, depending on the value of $h + J_2\Delta$, the spin transfer goes via either of the states $|\downarrow G_\pm \downarrow\rangle$.

Consider now $N_\uparrow = 2$ spin excitations. In the basis of states $\{|\uparrow\uparrow\downarrow\downarrow\downarrow\rangle, |\uparrow\downarrow\uparrow\downarrow\downarrow\rangle, |\uparrow\downarrow\downarrow\uparrow\downarrow\rangle, |\uparrow\downarrow\downarrow\downarrow\uparrow\rangle, |\downarrow\uparrow\uparrow\downarrow\downarrow\rangle, |\downarrow\uparrow\downarrow\uparrow\downarrow\rangle, |\downarrow\uparrow\downarrow\downarrow\uparrow\rangle, |\downarrow\downarrow\uparrow\uparrow\downarrow\rangle, |\downarrow\downarrow\uparrow\downarrow\uparrow\rangle, |\downarrow\downarrow\downarrow\uparrow\uparrow\rangle\}$, the Hamiltonian reads

$$H = -h\mathbb{I} + \begin{pmatrix} -J_1\Delta & -J_2 & 0 & 0 & 0 & 0 & 0 & 0 & 0 & 0 \\ -J_2 & 2h - 2h' + J_2\Delta & -J_2 & 0 & -J_1 & 0 & 0 & 0 & 0 & 0 \\ 0 & -J_2 & J_1\Delta & -J_1 & 0 & -J_1 & 0 & 0 & 0 & 0 \\ 0 & 0 & -J_1 & -2h' + (J_1 - J_2)\Delta & 0 & 0 & -J_1 & 0 & 0 & 0 \\ 0 & -J_1 & 0 & 0 & 2h & -J_2 & 0 & 0 & 0 & 0 \\ 0 & 0 & -J_1 & 0 & -J_2 & 2h' + (J_1 + J_2)\Delta & -J_1 & -J_2 & 0 & 0 \\ 0 & 0 & 0 & -J_1 & 0 & -J_1 & J_1\Delta & 0 & -J_2 & 0 \\ 0 & 0 & 0 & 0 & 0 & -J_2 & 0 & 2h & -J_1 & 0 \\ 0 & 0 & 0 & 0 & 0 & 0 & -J_2 & -J_1 & 2h - 2h' + J_2\Delta & -J_2 \\ 0 & 0 & 0 & 0 & 0 & 0 & 0 & 0 & -J_2 & -J_1\Delta \end{pmatrix}, \quad (39)$$

where $\mathbb{I}$ is the identity matrix. We prediagonalize the gate part to obtain the new basis $\{|\uparrow G_+ \downarrow\rangle, |\uparrow G_0 \downarrow\rangle, |\uparrow G_- \downarrow\rangle, |\uparrow\downarrow\downarrow\downarrow\uparrow\rangle, |\downarrow \bar{G}_- \downarrow\rangle, |\downarrow \bar{G}_0 \downarrow\rangle, |\downarrow \bar{G}_+ \downarrow\rangle, |\downarrow G_- \uparrow\rangle, |\downarrow G_0 \uparrow\rangle, |\downarrow G_+ \uparrow\rangle\}$, where $\bar{G}_{\pm,0}$ are obtained from $G_{\pm,0}$ via the replacement $\uparrow\leftrightarrow\downarrow$. We place the control spin excitation at the gate in one of the states $|G_{\pm,0}\rangle$ which is different from the one we chose via the magnetic field above for the resonant transfer. This spin then remains stationary since it cannot leave the gate due to the energy mismatch $\sim J_2 \gg J_1$. It also blocks the resonant transfer of the

target spin from site $j = 1$ to site $j = 5$, which cannot overcome the energy mismatch to the double excitation states $|\bar{G}_{\pm,0}\rangle$ of the gate.

Similar arguments can be used to construct a spin transistor with longer chains, as outlined in the main text.

Supplementary Note 4: implementation of the XXZ spin chain with strongly interacting atoms.

Here we outline a procedure[3–6] to map a system of strongly interacting atoms confined in a 1D trapping potential onto the Heisenberg XXZ spin model. To this end, we consider a two-component Bose gas of atoms. Denoting the components as spin-up and spin-down, the total number of atoms is $N = N_\uparrow + N_\downarrow$. The strong contact interaction between the atoms is modeled by the Dirac delta function potential, and, hence, the Hamiltonian is ($\hbar = 1$)

$$H_{atom} = \sum_{\sigma=\uparrow,\downarrow} \sum_{j=1}^{N_\sigma} \left[H_0(x_{\sigma,j}) + \frac{g_{\sigma\sigma}}{mL} \sum_{j'>j}^{N_\sigma} \delta(x_{\sigma,j} - x_{\sigma,j'}) \right] + \frac{g_{\uparrow\downarrow}}{mL} \sum_{i=1}^{N_\uparrow} \sum_{j'=1}^{N_\downarrow} \delta(x_{\uparrow,j} - x_{\downarrow,j'}) + \frac{1}{g} \sum_{\sigma=\uparrow,\downarrow} \sum_{j=1}^{N_\sigma} B(x_{\sigma,j}) \sigma_z^j, \quad (40)$$

where $H_0(x) = -\frac{1}{2m}\frac{\partial^2}{\partial x^2} + \frac{1}{mL^2} V(x/L)$ is the single-particle Hamiltonian in a one-dimensional trapping potential $V(x/L)$ with a characteristic length L, $x_{\sigma,j}$ is the coordinate of the jth particle with spin $\sigma = \{\uparrow, \downarrow\}$, m is the mass assumed equal for all particles, $B(x)$ is a spatially inhomogeneous magnetic field, and the Pauli σ_z^j operator acts on the spin of the jth particle. The interaction strengths are $g_{\uparrow\downarrow} = g_{\downarrow\uparrow} \equiv g > 0$ and $g_{\uparrow\uparrow} \equiv \kappa g$, where the parameter $\kappa > 0$ determines the interspecies interaction for bosonic atoms, while $\kappa \to \infty$ can be seen as the fermionic limit.

In general, the N-particle eigenstate of the system can be written as [3,4]

$$\Psi = \sum_k a_k \theta(x_{P_k(1)}, \ldots, x_{P_k(N)}) \Psi_0(x_1, \ldots, x_N), \quad (41)$$

where the summation runs over all $N!$ permutations P_k of coordinates, $a_k \in \mathbb{R}$ are the expansion coefficients which depend on the ordering of particles, $\theta(x_1, \ldots, x_i, \ldots, x_j, \ldots, x_N) = 1$ if $x_1 < x_2 < \cdots < x_i < \cdots < x_j < \cdots < x_N$ and zero otherwise. The function Ψ_0 is the fully antisymmetrized N-particle wavefunction, i.e., Slater determinant constructed from the single-particle solutions of the Schrödinger equation for a particle in the potential $V(x/L)$. As such, Ψ_0 describes the system of N identical fermions that has energy E_0. Note that E_0 is $M(N_\uparrow, N_\downarrow) = N!/(N_\uparrow! N_\downarrow!)$ fold degenerate, since the energy does not depend on the particle ordering. For small but finite $1/g$, the N-particle energy of the interacting system can be written in linear order in $1/g$ as [3,4]

$$E = E_0 - \frac{1}{g} \frac{\sum_{j=1}^{N-1} (A_j + \frac{2}{\kappa} C_j + \frac{2}{\kappa} D_j) \alpha_j}{\sum_{k=1}^{M(N_\uparrow, N_\downarrow)} a_k^2} + \frac{1}{g} \frac{\sum_k a_k^2 \sum_{\sigma=\uparrow,\downarrow} \sum_{j=1}^{N_\sigma} \langle \theta(x_{P_k(1)}, \ldots, x_{P_k(N)}) \Psi_0 | B(x_{\sigma,j}) \sigma_z^j | \Psi_0 \rangle}{\langle \theta \Psi_0 | \Psi_0 \rangle \sum_k a_k^2}, \quad (42)$$

where $A_j = \sum_{k=1}^{M(N_\downarrow-1,N_\uparrow-1)}(a_{j|k}-b_{j|k})^2$, $C_j = \sum_{k=1}^{M(N_\downarrow,N_\uparrow-2)} c^2_{j|k}$ and $D_j = \sum_{k=1}^{M(N_\downarrow-2,N_\uparrow)} d^2_{j|k}$. Here $a_{j|k}$ denote those coefficients a_k in the expansion in Supplementary Equation (41) for which $x_\uparrow$ is at position j followed by $x_\downarrow$ at position $j+1$. Similarly, coefficients $b_{j|k}$ correspond to $x_\downarrow$ at position j followed by $x_\uparrow$ at position $j+1$, coefficients $c_{j|k}$ correspond to $x_\uparrow$ at position j followed by $x_\uparrow$ at position $j+1$, and coefficients $d_{j|k}$ correspond to $x_\downarrow$ at position j followed by $x_\downarrow$ at position $j+1$.

The geometric factors α_j depend on both the total number of particles and the single-particle solutions of the Schrödinger equation for a particle in potential $V(x)$. An explicit expression for α_j reads

$$\alpha_j = \frac{1}{m^2}\frac{\int\prod_{i=1}^N \mathrm{d}x_i\theta(x_1,\ldots,x_N)\delta(x_1-x_j)(\partial\Psi_0)^2}{\int\prod_{i=1}^N \mathrm{d}x_i\theta(x_1,\ldots,x_N)|\Psi_0(x_1,\ldots,x_N)|^2}, \tag{43}$$

where $\partial\Psi_0 = \left(\frac{\partial\Psi_0}{\partial x_1}\right)_{x_1=x_N}$, i.e., one first takes the partial derivative of the non-interacting N-particle wave function Ψ_0 with respect to x_1 and then sets $x_1 = x_N$.

For strong interactions, $g \gg 1$, the Hamiltonian in Supplementary Equation (40) can be mapped onto the Heisenberg XXZ spin model Hamiltonian [4], cf. Supplementary Equation (1), with

$$\Delta = 1-\frac{2}{\kappa}, \tag{44}$$

$$J_j \equiv -\frac{\alpha_j}{g}, \tag{45}$$

$$h_j \equiv \frac{\int\prod_{i=1}^N \mathrm{d}x_i\theta(x_1,\ldots,x_N)|\Psi_0(x_1,\ldots,x_N)|^2 B(x_j)}{\int\prod_{i=1}^N \mathrm{d}x_i\theta(x_1,\ldots,x_N)|\Psi_0(x_1,\ldots,x_N)|^2}. \tag{46}$$

Hence, the shape of the one-dimensional confining potential will determine the exchange interaction coefficients J_j.

We note that the form of the resulting spin-chain Hamiltonian depends on our choice of the phase of $\theta(x_1,...,x_N)\Psi_0$. For instance, if in Supplementary Equation (41) we choose $|\Psi_0|$ instead of Ψ_0, then we obtain the spin-chain Hamiltonian of the form [5]

$$H_{|\Psi_0|} = \sum_{j=1}^N h_j\sigma_z^j - \frac{1}{2}\sum_{j=1}^{N-1} J_j[-\sigma_x^j\sigma_x^{j+1} - \sigma_y^j\sigma_y^{j+1} + \Delta\sigma_z^j\sigma_z^{j+1}]. \tag{47}$$

This Hamiltonian is related to H in Supplementary Equation (1) through the unitary transformation[7] which rotates the spins at every other site, i.e., $\sigma^i_{x,y} \to -\sigma^i_{x,y}$ for $i = 1,3,...$. This ambiguity in the phase choice does not affect any of the results presented in the main text, since the observables we calculate depend on the absolute values of a_k. However, we note that for bosonic systems the use of $H_{|\Psi_0|}$ instead of H can facilitate calculations of such quantities as correlations functions etc., since $|\Psi_0|$ is symmetric with respect to two-body exchanges. Other choices of the phase in Supplementary Equation (41), e.g., $\Psi_0 \to e^{i\phi_k}\Psi_0$, $\phi_k \in \mathbb{R}$, lead to other spin-chain Hamiltonians that are unitarily equivalent.

For conditional spin transfer in a four-particle spin chain, we require that the exchange coefficients satisfy $J_1 = J_3$ and $J_1/J_2 \ll 1$, which can be realized in a symmetric triple-well potential $V(x)$ given by

$$V(x) = -V_0\left[e^{-a(x-x_0)^2} + e^{-a(x+x_0)^2}\right] - Ue^{-bx^2}, \tag{48}$$

where V_0 and U are in units of $\varepsilon = \frac{1}{mL^2}$ ($\hbar = 1$). We set the centers of the spatial Gaussians with $x_0 = \frac{7L}{16}$ and choose the constants $a = \frac{384}{L^2}$ and $b = \frac{64}{5L^2}$. Such a potential can be realized in an optical trap using appropriately focused, far-off-resonant laser beams [8].

The shape of the potential for values $V_0 = 500$ and $U = 200$ is shown in Fig. 2a of the main text. The potential wells next to the edges are very deep compared to the broad well in the middle. The single-particle wavefunctions are also schematically shown there. We observe that the two lowest-energy eigenfunctions have nearly degenerate energies and are located almost fully in the deep wells next to the boundaries of the potential, which translates into small exchange couplings $J_{1,3}$. The higher energy wavefunctions are mostly in the shallow middle well, and their large energy splitting corresponds to strong exchange coupling J_2. In Figs. 2b and 2c of the main text, we show the ratio of exchange coefficients J_2/J_1 as a function of the parameters U and V in the potential in Supplementary Equation (48). This ratio can reach rather large values, for example $J_2/J_1 > 10$ for $U = 150 - 550$ at $V_0 = 500$. Therefore, this potential can be used to attain necessary parameters of the XXZ model Hamiltonian to realize conditional spin transfer.

Supplementary References

1. Tao, D. & Yasuda, M. A Spectral Characterization of Generalized Real Symmetric Centrosymmetric and Generalized Real Symmetric Skew-Centrosymmetric Matrices. *SIAM J. Matrix Anal. Appl.* **23,** 885–895 (2002).

2. Nield, D. A. Odd-Even Factorization Results for Eigenvalue Problems. *SIAM Rev.* **36,** 649–651 (1994).

3. Volosniev, A. G., Fedorov, D. V., Jensen, A. S., Valiente, M. & Zinner, N. T. Strongly interacting confined quantum systems in one dimension. *Nat. Commun.* **5,** 5300 (2015).

4. Volosniev, A. G. *et al.* Engineering the dynamics of effective spin-chain models for strongly interacting atomic gases. *Phys. Rev. A* **91,** 023620 (2015).

5. Deuretzbacher, F., Becker, D., Bjerlin, J., Reimann, S. M. & Santos, L. Quantum magnetism without lattices in strongly interacting one-dimensional spinor gases. *Phys. Rev. A* **90,** 013611 (2014).

6. Levinsen, J., Massignan, P., Bruun, G. M. & Parish, M. M. Strong-coupling ansatz for the one-dimensional Fermi gas in a harmonic potential. *Sci. Adv.* **1,** e1500197 (2015).

7. Takahashi, M. *Thermodynamics of One-Dimensional Solvable Models* (Cambridge Univ. Press, 1999).

8. Murmann, S. *et al.* Two Fermions in a Double Well: Exploring a Fundamental Building Block of the Hubbard Model. *Phys. Rev. Lett.* **114,** 080402 (2015).
